# Ly6C^hi^ Monocyte Recruitment Is Responsible for Th2 Associated Host-Protective Macrophage Accumulation in Liver Inflammation due to Schistosomiasis

**DOI:** 10.1371/journal.ppat.1004282

**Published:** 2014-08-21

**Authors:** Marcia Nascimento, Stanley C. Huang, Amber Smith, Bart Everts, Wing Lam, Elizabeth Bassity, Emmanuel L. Gautier, Gwendalyn J. Randolph, Edward J. Pearce

**Affiliations:** 1 Department of Pathology and Immunology, Washington University School of Medicine, St. Louis, Missouri, United States of America; 2 Trudeau Institute, Saranac Lake, New York, United States of America; University of York, United Kingdom

## Abstract

Accumulation of M2 macrophages in the liver, within the context of a strong Th2 response, is a hallmark of infection with the parasitic helminth, *Schistosoma mansoni*, but the origin of these cells is unclear. To explore this, we examined the relatedness of macrophages to monocytes in this setting. Our data show that both monocyte-derived and resident macrophages are engaged in the response to infection. Infection caused CCR2-dependent increases in numbers of Ly6C^hi^ monocytes in blood and liver and of CX_3_CR1^+^ macrophages in diseased liver. Ly6C^hi^ monocytes recovered from liver had the potential to differentiate into macrophages when cultured with M-CSF. Using pulse chase BrdU labeling, we found that most hepatic macrophages in infected mice arose from monocytes. Consistent with this, deletion of monocytes led to the loss of a subpopulation of hepatic CD11c^hi^ macrophages that was present in infected but not naïve mice. This was accompanied by a reduction in the size of egg-associated granulomas and significantly exacerbated disease. In addition to the involvement of monocytes and monocyte-derived macrophages in hepatic inflammation due to infection, we observed increased incorporation of BrdU and expression of Ki67 and MHC II in resident macrophages, indicating that these cells are participating in the response. Expression of both M2 and M1 marker genes was increased in liver from infected vs. naive mice. The M2 fingerprint in the liver was not accounted for by a single cell type, but rather reflected expression of M2 genes by various cells including macrophages, neutrophils, eosinophils and monocytes. Our data point to monocyte recruitment as the dominant process for increasing macrophage cell numbers in the liver during schistosomiasis.

## Author Summary

Schistosomiasis is an important neglected tropical disease caused by parasitic worms of the genus *Schistosoma*. During infection with *S. mansoni*, parasite eggs become trapped in the liver and elicit granulomatous inflammation characterized by accumulations of immune cells intermixed with liver cells around the eggs. This inflammation is responsible for disease symptoms, but also plays an important role in protecting the host against liver damage that can be caused by egg products. Granulomas, by definition, contain a significant number of macrophages (phagocytic cells of the immune system). Recent work has emphasized that macrophage numbers in inflammation can increase due either to recruitment of precursor cells (called monocytes) from the blood, or as a result of proliferation of tissue-resident macrophages. Local proliferation has been noted in other worm infections, during which the immune response is Th2-like and IL-4 produced by Th2 cells promotes macrophages to become “alternatively (or M2) activated”. We examined the origin of the increased numbers of macrophages in liver inflammation due to schistosomiasis, in which there is also a prominent Th2 response. We found that the cells mostly originated from monocytes recruited into the tissue from the blood. This response was critical for host survival during infection.

## Introduction

Schistosomiasis is a complex multiorgan disease caused by infection with helminth parasites of the genus *Schistosoma*, and characterized by the development of granulomatous lesions around parasite eggs trapped within organs such as the liver and intestine. Granulomas are, by definition, macrophage-rich, but in schistosomiasis are recognized to contain substantial numbers of additional cells such as eosinophils, reflecting the fact that infection is associated with the development of a marked Th2 response [1].

In general, expanded macrophage populations in inflammatory sites are thought to arise from the recruitment of circulating inflammatory (Ly6C^h^) monocytes from the bloodstream, and the *in situ* differentiation of these cells into macrophages [2]. Mobilization of monocytes from bone marrow is dependent on the interactions of CCL2 and CCL7 with CCR2, and can be promoted by type I interferons and TLR agonists [3]. Tissue-recruitment of monocytes is documented in viral, bacterial, fungal and protozoal infections [4], where monocytes that have entered tissues assume the characteristics of M1 macrophages, or TIP dendritic cells, both of which make TNFα and NO [3]. In contrast, little is known about the contribution of monocytes to inflammation during schistosomiasis. This is an interesting issue because recent reports have stressed that during Th2-dominated responses to nematode helminth parasites, macrophage rich inflammatory infiltrates arise as a result of IL-4-driven local macrophage proliferation, and not monocyte recruitment [5], [6]. A major effect of IL-4 on macrophages is to induce a program of gene expression that defines the M2 phenotype [7]. M2 macrophages are implicated in wound healing, metabolic homeostasis of adipose tissue, and in protective immunity to helminths, where type 2 immunity dominates [8], [9]

Schistosomiasis is a chronic infection. However, in the absence of IL-4, or IL-4Rα, the infection is acutely lethal [10]–[12]. The protective functions of IL-4 are considered to be macrophage-dependent, since infected IL-4Rα^fl/fl^LysMCre mice develop the same lethal acute disease as infected *Il4* ^−/−^ mice [12]. Arginase 1 and Relmα, expression of both of which by macrophages is induced by IL-4/IL-13, are implicated in protective effects of M2 cells in schistosomiasis [13]–[15]. The absence of M2 macrophages during acute infection is associated with the development of cachexia with elevated levels of TNFα and NO, suggesting that commitment to the M2 pathway of activation contributes to the suppression or prevention of proinflammatory mediator production [10]. Other evidence supports the idea that M2 macrophages are playing a regulatory role in schistosomiasis, not only limiting inflammation but also preventing excessive tissue remodeling [14]. Nevertheless, despite the importance of macrophages in schistosomiasis, little is known of the origin of these cells in the liver during infection. Here we set out to establish whether the macrophage population hyperplasia in this setting results from monocyte recruitment or *in situ* proliferation. Our data suggest that a small percentage of resident macrophages in the liver are in cell cycle during infection, but that monocyte recruitment dominates as the mechanism for increasing macrophage numbers in this setting.

## Results

**Figure 1:**
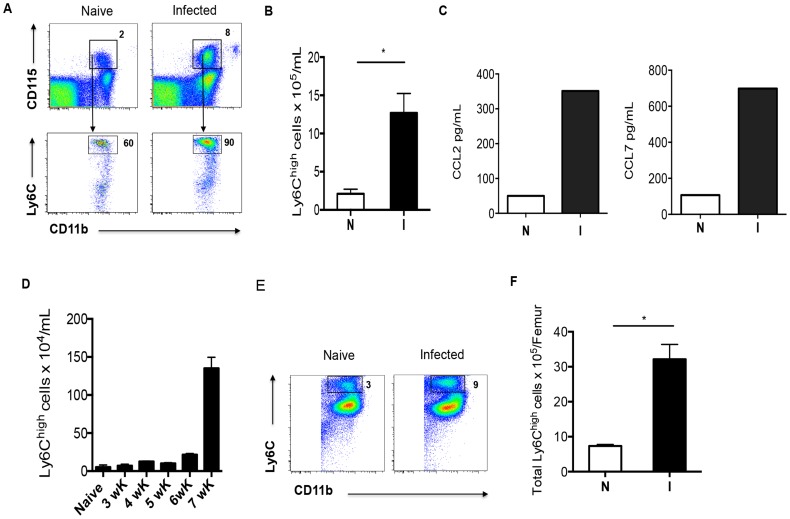
Schistosomiasis induces monocytosis. Ly6C^hi^ and Ly6C^lo^ monocytes were identified in the blood and bone marrow by flow cytometry, and enumerated by back-calculation from nucleated cell counts. **A**. The Ly6C^hi^ CD115^+^ CD11b^+^ population of monocytes expands in the peripheral blood of 7 week infected mice. Numbers outside boxed areas indicate percentages of total indicated populations. **B**. The number of Ly6C^hi^ monocytes circulating in infected mice (I) is significantly greater than in naïve mice (N). **C**. Plasma concentrations of CCL2 and CCL7. **D**. Kinetics of increase in Ly6C^hi^ monocyte numbers in the blood during infection. **E**. Increased contribution of Ly6C^hi^ monocytes to the CD11b^+^ compartment within the bone marrow as a result of infection. **F**. Numbers of Ly6C^hi^ monocytes in the bone marrow of naïve (N) and infected (I) mice. Data in A and E are concatenated from samples from 3 to 5 mice per group. Graphs B, D and F present mean ± SEM of three to five mice/group. *P* values of 0.05 or less are marked by an asterisk. Data are representative of findings from at least 3 experiments, except for C, where pooled samples from 5 mice per group were measured once.

To begin to explore whether monocytes give rise to macrophages in inflamed hepatic tissues, we first examined monocyte numbers in the blood of naïve vs. infected mice. We found that infected mice developed a monocytosis, evident as increased numbers of Ly6C^hi^ CD11b^+^CD115^+^ cells in the blood at week 7 of infection (Figs. 1A, B). This is consistent with elevated plasma levels of the chemokines CCL2 and CCL7 (Fig. 1C), the ligands for CCR2 (expressed on Ly6C^hi^ cells, not on Ly6C^lo^ monocytes [16]), that are responsible for mobilizing monocytes from bone marrow and splenic reservoirs into the circulation. We next asked when during infection monocytosis develops. We reasoned that increases in blood monocyte numbers at the time when egg-induced tissue inflammation is initiated would be consistent with the contribution of monocytes to the inflammatory process. We found that the number of monocytes in the blood of infected mice increased between weeks 5 and 7 of infection (Fig. 1D), correlating with the time of onset of parasite egg production [17]. Increased monocyte numbers in the blood were associated with increased monopoiesis, detected as an increased frequency and number of Ly6C^hi^ monocytes in the bone marrow (Fig. 1E, F). Similarly coordinated increases in blood and bone marrow monocyte numbers have been seen in mice infected with *L. monocytogenes* [4].

**Figure 2:**
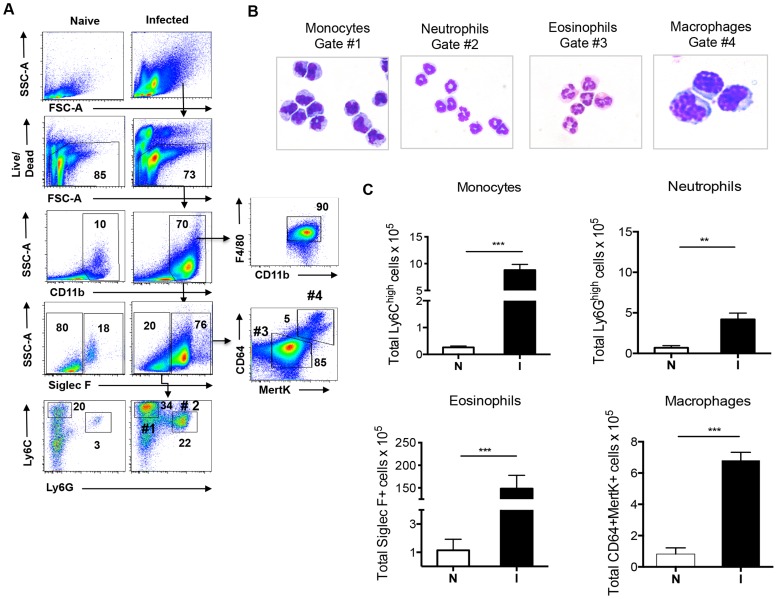
Hepatic inflammation during infection is associated with increased numbers of monocytes, neutrophils, eosinophils and macrophages. **A**. The gating strategy for identifying and sorting monocytes, neutrophils, eosinophils and macrophages from leukocyte populations isolated from the livers of 7 week infected mice; data from naïve mice are shown for comparison. Numbers inside or outside boxed areas indicate percent cells. #1: monocytes. #2: neutrophils. #3 eosinophils. #4: macrophages. **B**. Images of cytospins of cells from gates 1–4. **C**. Numbers of indicated cells in the livers of naïve (N) and infected (I) mice, as enumerated by flow cytometry and back-calculation from counts of nucleated cells. Data are representative of five independent experiments. Graphs present the mean ± SEM of five mice/group. **P<0.01 and *** *P*<0.001, as determined by two-tailed *t* test.

If macrophages in diseased liver due to schistosomiasis are derived from monocytes, we would expect to detect extravasated monocytes within this organ. We used flow cytometry to characterize the cellular infiltrate in the liver, and, as anticipated from the histopathological picture [18], the infiltrate was complex. Using the gating strategy depicted in Fig. 2A, we were able to identify monocytes within the liver, and discovered a significant increase in this population as a result of infection (Fig. 2A, B, gate #1). The infiltrate was also rich in neutrophils (Fig. 2A, B, gate #2) and eosinophils (Fig. 2A, B, gate #3). To define macrophages we took advantage of recent findings that co-expression of the tyrosine kinase Mer (MerTK) and CD64 provides a robust marker for macrophages in the liver [19]. F4/80 was not a sufficient marker of macrophages in this setting, since it also marked eosinophils and monocytes (Fig. 1A, and [19]). In our initial experiments we found that the MerTK^+^CD64^+^ cells fell within a Siglec-F^+^ gate (Fig. 2A, gate #4). Siglec-F is an accepted marker for eosinophils, but it is also highly expressed on alveolar macrophages [20]. It clearly additionally marks macrophages within the liver during schistosome infection. We were able to identify a distinct MerTK^+^CD64^+^ macrophage population within the Siglec-F^+^ gate (Fig. 2A, B, gate #4). Enumeration of cells within gates 1–4 revealed significant increases in all populations as a result of infection (Fig. 2C).

**Figure 3:**
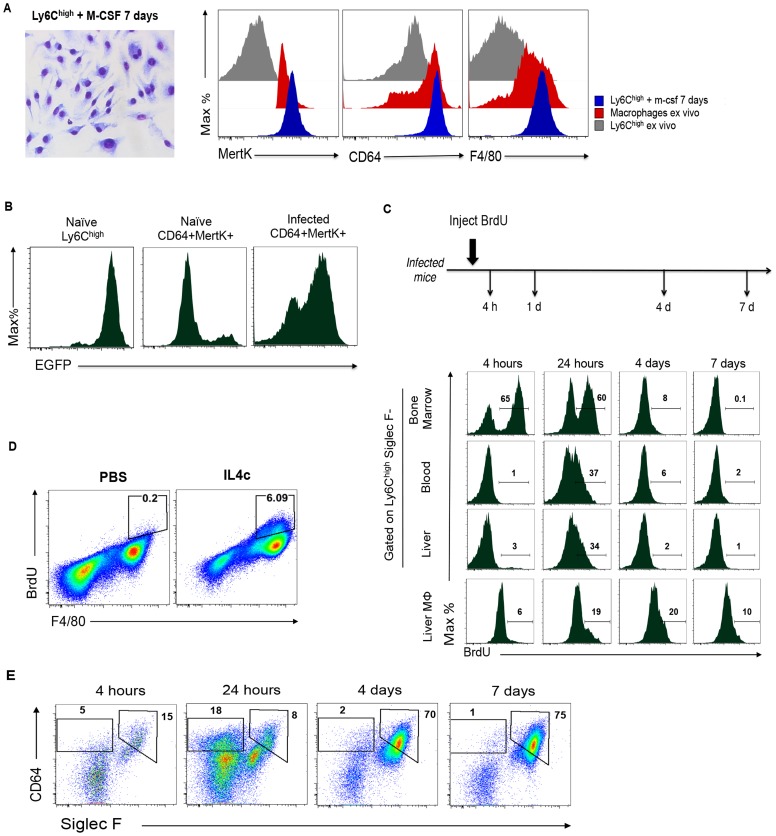
Monocytes give rise to macrophages within the liver during infection. **A**. Ly6C^hi^ monocytes sorted from liver of infected mice differentiate into macrophages following culture with M-CSF: a cytospin of macrophages grown in this way is shown (left panel) along with flow cytometric analysis of the same cells vs. macrophages or Ly6C^hi^ monocytes sorted from infected liver (*ex vivo*) for expression of MertK and CD64 (right panel). This experiment was repeated three times with similar results. **B**. The expression of GFP in blood monocytes (Ly6C^hi^ CD11b^+^) from naïve mice, and on CD64^+^MertK^+^ macrophages from naïve or infected *C*x*_3_cr1^gfp/+^* mice. Data are from cells pooled from 3 individual mice, and are representative of two independent experiments. **C**. BrdU is rapidly incorporated into monocytes and appears in macrophages only later following initiation of labeling. Infected mice were injected with BrdU and bone marrow, blood, and hepatic leukocytes collected at the times shown were pooled and monocytes and macrophages (Mphi) were stained for incorporated BrdU. Results are data from one independent experiment with three to five mice per group, and are representative of 3 independent experiments. **D**. BrdU incorporation into peritoneal macrophages in mice injected i.p. with IL-4/anti-IL-4 complexes. Data are concatenated from 3 mice per group and are representative of 2 independent experiments. **E**. Within the entire BrdU labeled hepatic leukocyte population, CD64^+^ Siglec-F^hi^ macrophages initially represent only a small percentage of all cells, but over time post-labeling come to represent 75% of the labeled population (by day 7). In these plots, monocytes fall within the indicated CD64^int^ Siglec-F^Neg^ gate; these data are from the experiments described in C.

To explore the possibility that the expanded population of hepatic macrophages associated with egg-induced inflammation largely results from the recruitment of monocytes rather than *in situ* proliferation of resident cells, we first asked whether sort-purified monocytes from liver tissues of infected mice were capable of differentiating into macrophages in response to M-CSF *in vitro* [21]. M-CSF is readily detectable in the serum of infected mice, where it is present at similar levels (7.9 ng/ml) as in naïve mice (6.6 ng/ml). After 7 days of culture with M-CSF, the cells had acquired a macrophage like appearance and expressed high levels of MerTK, CD64 and F4/80 equivalent to macrophages sorted from diseased liver (Fig. 3A). Next, we determined whether macrophages in the liver express CD115 or CX_3_CR1, markers that define blood monocytes [2], [22]. We found that CD115 was lost on monocytes that we recovered from the liver and therefore could not be used to trace their relationship to macrophages (data not shown). In contrast, CX_3_CR1, reported by GFP in *Cx3cr1^gfp/+^* mice, was expressed by all Ly6C^hi^ monocytic cells in the liver of infected *Cx3cr1^gfp/+^* mice (data not shown), and in these animals the majority of hepatic MerTK^+^CD64^+^ cells were also GFP^+^ (Fig. 3B), indicating a significant contribution of monocytes to the macrophage pool during infection. In contrast, very few MerTK^+^CD64^+^ cells in naïve *Cx3cr1^gfp/+^* mice were GFP^+^ (Fig. 3B). These data are consistent with a previous report that GFP-positive macrophages could be isolated from the livers of schistosome infected *Cx3cr1* reporter mice [23].

In other settings of type 2 immunity due to helminths, macrophages have been reported to robustly incorporate BrdU *in situ* within 3 hours of BrdU injection as they proliferate in response to IL-4 [5]. We asked whether macrophages were proliferating in the liver during schistosomiasis by injecting mice with BrdU and then using flow cytometry to detect BrdU in MerTK^+^CD64^+^ macrophages at times thereafter. We additionally investigated BrdU incorporation into monocytes. The pulse-chase experimental design allowed us to effectively identify cells proliferating at the time of BrdU injection, and then to ascertain whether cells labeled within this period had differentiated into other cell types, not labeled within the first 4 h, but which were BrdU^+^ at later times (Fig. 3C). In this way the lineage relatedness of monocytes to macrophages could be defined. By 4 h after BrdU injection, 65% of monocytes in the bone marrow were labeled, a percentage that was approximately twice that observed in naive mice (data not shown). Labeled monocytes were not observed in the blood or liver at this time (Fig. 3C). However, approximately 6% of macrophages in the liver were BrdU^+^ (Fig. 3C), a percentage that was higher than in naïve mice (where <%2 of macrophages, defined by the same criteria, were positive for BrdU at this time point, data not shown). This percentage of macrophages that labeled within 4 h was similar to that observed after a 4 h BrdU pulse label in peritoneal macrophages of mice that had been injected i.p. with IL-4/anti-IL-4 complexes (Fig. 3D, and [5]). At 4 h after labeling, few BrdU^+^ monocytes were evident in the blood or liver (Fig. 3C). By 24 h approximately 35% of the monocytes in the blood and liver were BrdU^+^, reflecting the exit of labeled monocytes from the bone marrow into the circulation, and their entry into hepatic tissues (Fig. 3C). At this time, approximately 20% of macrophages in the liver were BrdU^+^. By 4–7 days after injection, labeled monocytes were rare in the bone marrow, blood and liver, but BrdU^+^ hepatic macrophages remained detectable, albeit in numbers that were decreasing within this timeframe (Fig. 3C). Nevertheless, when we gated on all BrdU^+^ bone marrow derived (CD45^+^) cells within the liver at the various time points of the experiment, we found that by days 4–7 post labeling, 75% of all label-retaining cells could be categorized as macrophages (Fig. 3E). Our conclusion from these studies is that the majority of macrophages in the liver under these conditions are derived from monocytes that have entered from the blood.

**Figure 4:**
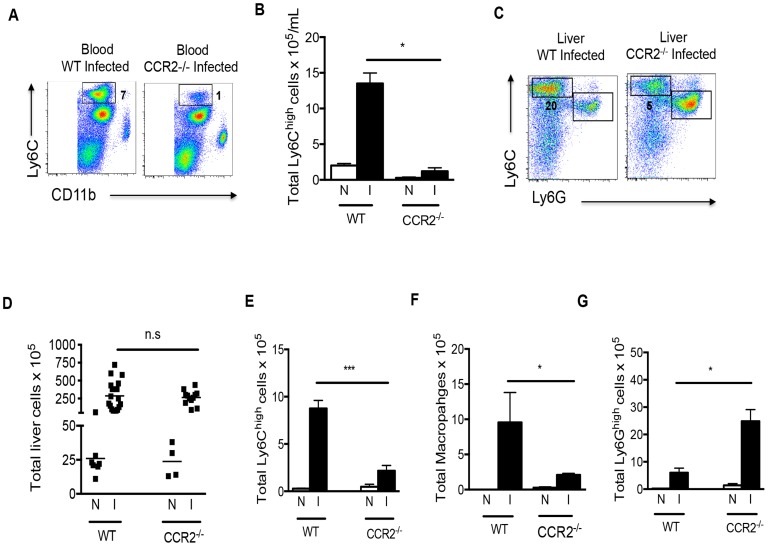
Infection induced monocytosis and increases in macrophage numbers within the liver are CCR2-dependent. The frequency (**A**) and overall number (**B**) of Ly6C^hi^ monocytes in the blood of infected *Ccr2^−/−^* mice is significantly lower than in infected WT mice. Numbers beside boxed areas indicate percent of total. **C**. The frequency of Ly6C^hi^ monocytes in the hepatic infiltrate is reduced in the absence of CCR2, while the frequency of Ly6G^hi^ cells increases. Numbers outside boxed areas indicate percent of total. **D**. Overall increases in hepatic leukocyte numbers due to infection in WT and *Ccr2^−/−^* mice are similar (N: naïve, I: infected); each point represents data from an individual mouse. Total numbers of Ly6C^hi^ monocytes (**E**), macrophages (**F**) and neutrophils (**G**) in the hepatic infiltrate of infected (I) vs. naïve (N) WT and *Ccr2^−/−^* mice. Cells gated as in Fig. 2 2. The data in A and C are concatenated from 3 mice per group. Data in B, D, E, F and G present the mean ± SEM of three or more mice/group. Data in all panels are representative of four independent experiments. *P<0.05 and *** *P*<0.001.

Recruitment of Ly6C^hi^ monocytes to inflammatory sites is dependent on CCR2 [24], which is strongly expressed by these cells. We found that, compared to infected WT mice, the frequency and number of Ly6C^hi^ monocytes in the blood (Fig. 4A, B) and the frequency of Ly6C^hi^ monocytes in the liver (Fig. 4C) during infection in *Ccr2^−/−^* mice was greatly diminished. Strikingly, macrophage numbers in livers of infected *Ccr2^−/−^* mice were significantly reduced as well (Fig. 4F), supporting the hypothesis that macrophages in diseased liver arise from infiltrating monocytes. Nevertheless, the magnitude of the overall hepatic infiltrate in infected *Ccr2^−/−^*mice was similar to that in WT mice (Fig. 4D). This was accounted for by the fact that monocytes (Fig. 4E) and macrophages (Fig. 4F) were replaced by a marked increase in the number of Ly6G^+^ neutrophils in the absence of CCR2 (Fig. 4G). We noted little difference between these two strains in the infection-induced eosinophilia (data not shown). This picture, revealed by flow cytometry, was confirmed by histopathology (data not shown).

Given the reported importance of macrophages for survival in schistosomiasis, we were intrigued that infected *Ccr2^−/−^* mice survived despite a relative failure to increase liver macrophage numbers. However, in repeated experiments we noted that *Ccr2^−/−^* mice appeared sick, as indicated by immobility, huddling and piloerection between weeks 6 and 7 post infection (not shown). This was not accompanied by weight loss and while infected mice were sacrificed between weeks 7 and 9 post infection for the experiments shown, we found that infected *Ccr2* ^−/−^ mice would survive into chronic infection if permitted (data not shown).

**Figure 5:**
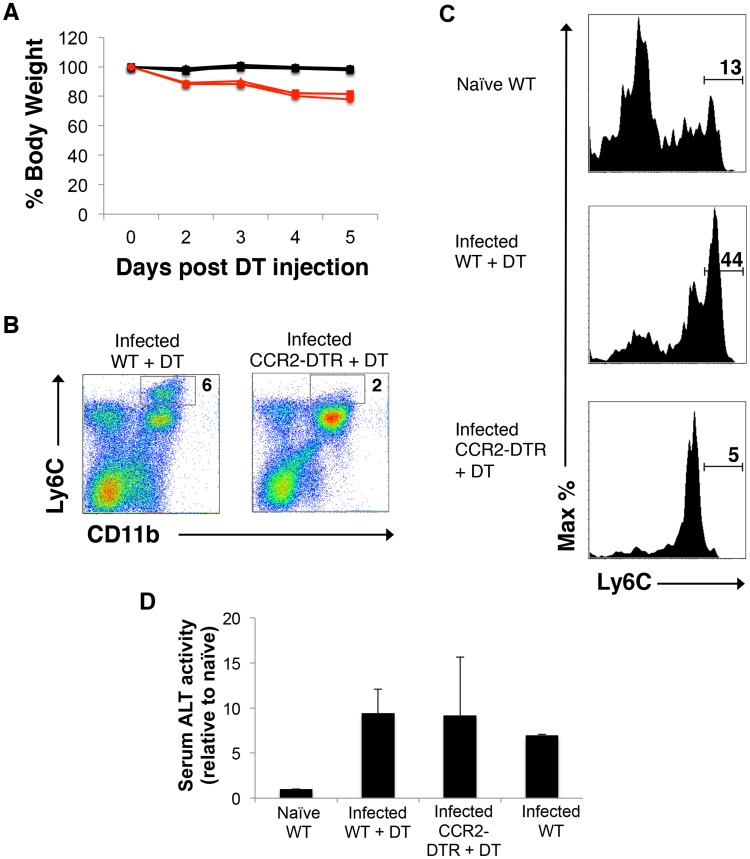
Depletion of monocytes in infected mice leads to severe acute weight loss. Infected wild type (WT) and CCR2-DTR mice were treated with DT beginning at week 6 of infection, weighed intermittently thereafter, and bled on the 5^th^ day following initiation of the treatment. **A**. Weight loss, shown as a percentage of the starting weight. Data from 3 individual mice per group are shown. **B**. Expression of Ly6c and CD11b on mononuclear blood cells, as measured by flow cytometry. Numbers represent percentages of total cells that fall within the indicated gates. Data are from pooled bleeds from 3 mice. Data in both panels are from one experiment, and representative of 2 independent experiments. **C**. The frequency of Ly6C^hi^ monocytes within the liver is increased as a result of infection, and reduced in CCR2-DTR mice following treatment with DT. Monocytes were identified by flow cytometry by sequential gating of live cells that were Siglec-F^NEG^, MerTK^NEG^, CD64^LO/INT^, CD11b^+^, F4/80^+^ and (as shown) Ly6c^HI^. Monocytes in livers of naïve WT mice, infected WT mice treated with DT, and infected CCR2-DTR mice treated with DT, as indicated. The monocyte gate is indicated and numbers refer to percentages of F4/80^+^ cells that fall within that gate. Data are from one experiment, representative of 2 independent experiments. **D**. Serum ALT levels. ALT activity in serum naïve mice was set to one, and fold increases in activity in serum from infected mice, as indicated, are shown. For the experimental groups, data are mean ± SD of values from 3 individual mice.

To further explore the importance of monocyte-derived macrophages in schistosomiasis, we examined the outcome of infection in CCR2-Diphtheria Toxin Receptor (DTR) mice, in which cells that express CCR2 also express DTR and can be acutely deleted by the injection of DT [25]. We injected groups of infected CCR2-DTR and wild type (WT) mice with DT at week 6 of infection, which is just prior to the increase in blood monocyte numbers (Fig. 1D). This treatment resulted in rapid weight loss in CCR2-DTR mice but not WT mice, such that by day 4 post injection CCR2-DTR mice had lost approximately 20% of their body weight (Fig. 5A); the mice succumbed from infection by day 8 post injection. Naïve CCR2-DTR mice treated with DT remained healthy and did not lose any weight over the time period of the study (data not shown). To analyze the effects of CCR2^+^ cell depletion, we sacrificed mice at day 5 post initiation of DT treatment, and used flow cytometry to assess blood monocyte levels. As expected, we found that CD11b^+^Ly6C^hi^ cells, which were also CD115^+^ (not shown), were lost in infected CCR2-DTR mice following DT treatment (Fig. 5B). DT treatment also depleted LyC^hi^ monocytes from the livers of infected CCR2-DTR mice (Fig. 5C). Serum levels of the liver enzyme alanine aminotransferase (ALT), a marker of hepatocellular injury, were no higher in DT treated infected CCR2-DTR mice than in DT treated infected WT mice, and DT treatment *per se* did not lead to liver damage in infected WT controls, since serum ALT levels in these mice were no higher than in untreated infected mice (Fig. 5D).

**Figure 6:**
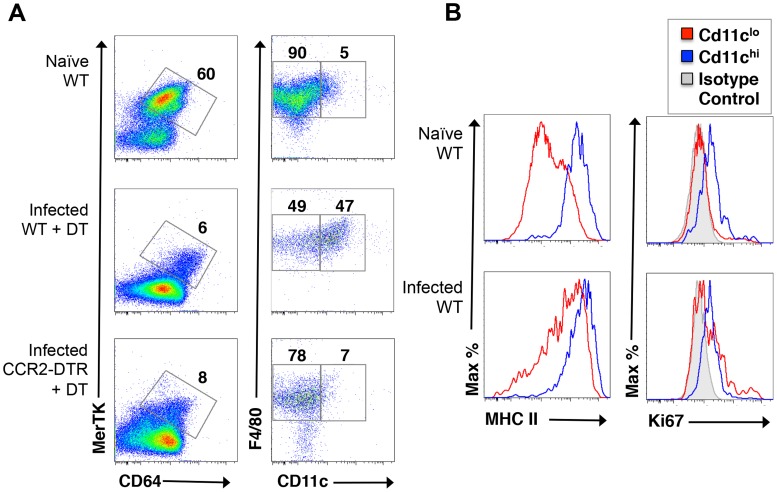
Monocyte depletion leads to the loss of a population of CD11c^hi^MHCII^hi^ macrophages from the livers of infected mice. **A**. Live cells isolated from the livers of naïve WT mice, or infected WT mice or infected CCR2-DTR mice treated with DT, were stained with antibodies against MerTK, CD64, F4/80 and CD11c and flow cytometry was used to measure expression of F4/80 and CD11c on MerTK^+^CD64^+^ cells. Numbers outside gated areas indicate percent of total. **B**. MHC II expression and Ki67 expression in CD11c^lo^ and CD11c^hi^ macrophages (defined as in A) were measured by flow cytometry.

We next used flow cytometry to assess the effect of acutely deleting monocytes on hepatic macrophage populations in infected mice. We found no overall change in the frequency of MerTK^+^CD64^+^ cells in DT treated infected CCR2-DTR mice compared to DT treated WT mice (Fig. 6A), although there were substantially fewer hepatic leukocytes following monocyte depletion (data not shown). However, when we further examined MerTK^+^CD64^+^ macrophages for expression of F4/80 and CD11c, which can be used to distinguish subsets of macrophages within tissues [19], we found that a population of F4/80^+^CD11c^hi^ cells was markedly increased as a result of infection and depleted by DT treatment in infected CCR2-DT mice (Fig. 6A). The CD11c^hi^ macrophages additionally expressed high levels of MHC II (Fig. 6B). We used Ki67 staining to broadly identify cycling cells in the resident CD11c^lo^ macrophages and monocyte-derived CD11c^hi^ macrophages. We found that Ki67 expression was increased in the CD11c^lo^ population in infected vs. naïve mice (Fig. 5B), suggesting that resident macrophages are responding to infection, a conclusion further supported by the fact that MHC II expression is increased on this population in infected mice (Fig. 6B). In our analyses, CD11c^hi^ macrophages were also positive for Ki67 (Fig. 6B), suggesting that these cells were proliferating, however this was true whether the cells were from naïve or infected mice, suggesting perhaps that this is an intrinsic property of these monocyte-derived cells (Fig. 6B).

**Figure 7:**
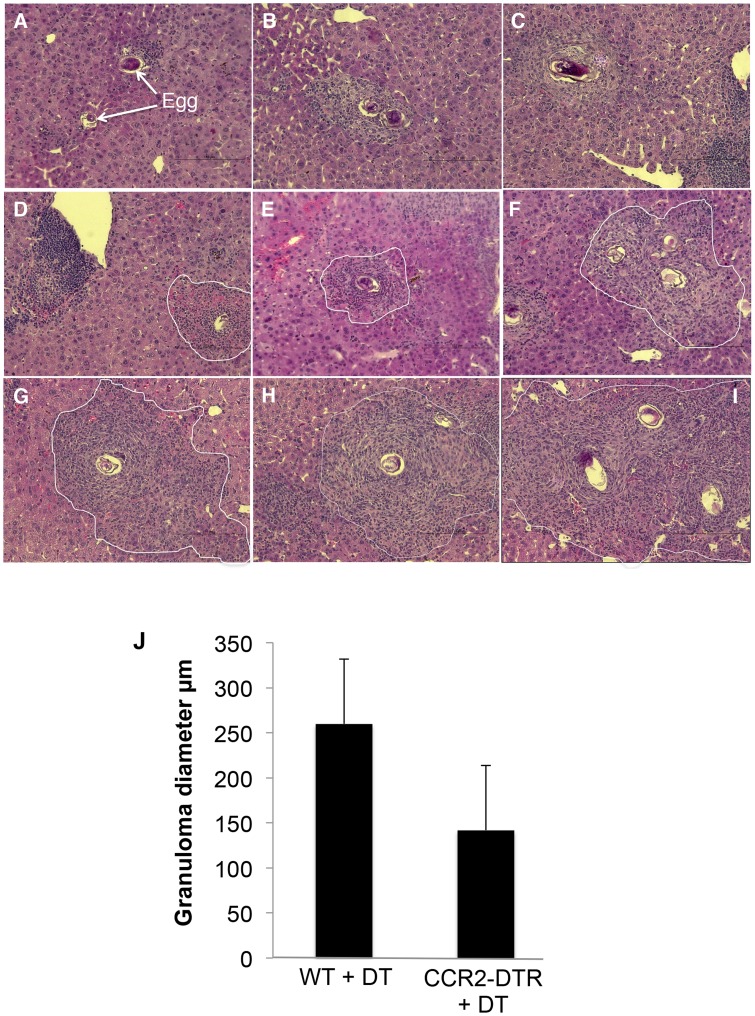
Depletion of monocytes results in reduced granuloma formation. Representative granulomas from acutely infected, DT treated CCR2-DTR (**A**–**F**) and WT mice (**G**–**I**). All images were photographed at 40×. In D – I, granulomas are delimited with a white line. Parasite eggs are clearly visible within each image (identified in panel **A** for orientation). **J**. Granuloma diameter. Bars are mean values ± SD of the diameters of 15 granulomas measured in liver sections from 3 infected mice (5 granulomas per mouse). The difference in size between the two groups is statistically significant at p<0.002 (Student's t test).

We used histology to assess whether deletion of monocytes had an effect on hepatic granuloma formation. We found that DT treatment in infected CCR2-DTR mice resulted in significantly reduced granulomatous inflammation (Fig. 7). Many eggs in these mice were associated with little or no inflammation (Fig. 7A), a situation observed only rarely in infected WT mice. Moreover, when granulomas were apparent in DT-treated CCR2-DTR mice (Fig. 7B–F), they were significantly smaller than those in infected DT-treated WT mice (Fig. 7G,H,I,J). We also noted marked accumulations of cells close to blood vessels in infected DT treated CCR2-DTR mice that were not apparent in infected DT-treated WT mice (Fig. 7D); the majority of cells in these accumulations were lymphocytic in appearance.

Disease progression marked by weight loss in DT treated infected CCR2-DTR mice was similar to that reported for infected IL-4^−/−^ and IL-4Rα^−/−^ mice [10]–[12], raising the possibility that depletion of monocytes had secondary effects on the Th2 cell response, with a resultant reduction in IL-4/IL-13 production. To examine this possibility, we prepared splenocytes and measured cytokine production following restimulation with soluble schistosome egg antigen (SEA). We found that the production of IFNγ, IL-5, IL-10 and IL-13 were largely similar between DT treated WT and CCR2-DTR mice (Table 1), although there was a trend towards greater production of IL-5 and IL-13, and lower production of IL-10 in the absence of CCR2^+^ cells. Strikingly, the only marked difference observed was in IL-4 production, where levels of this cytokine were higher in culture supernatants of splenocytes from DT treated CCR-DTR vs. WT mice. IL-17 levels were below the level of detection in our assay.

**Table 1:** Cytokine production by SEA-stimulated spleen cells.

	IFN-γ^2^	IL-4	IL-5	IL-13	IL-10
**CCR2-DTR** 1 **No stimulation**	0 (1)^3^	13 (7)	15 (10)	138 (138)	70 (67)
**CCR2-DTR +SEA**	23 (8)	705 (596)	399 (92)	819 (176)	718 (546)
**WT No stimulation**	1 (1)	10 (0)	5 (0)	8 (3)	7 (4)
**WT+SEA**	33 (16)	10 (0)	276 (119)	672 (123)	1264 (311)

1 CCR2-DTR and WT mice were treated with DT.

2 Cytokine amounts are in pg/ml.

3 Values are means (SD) for values from three mice assayed individually.

**Figure 8:**
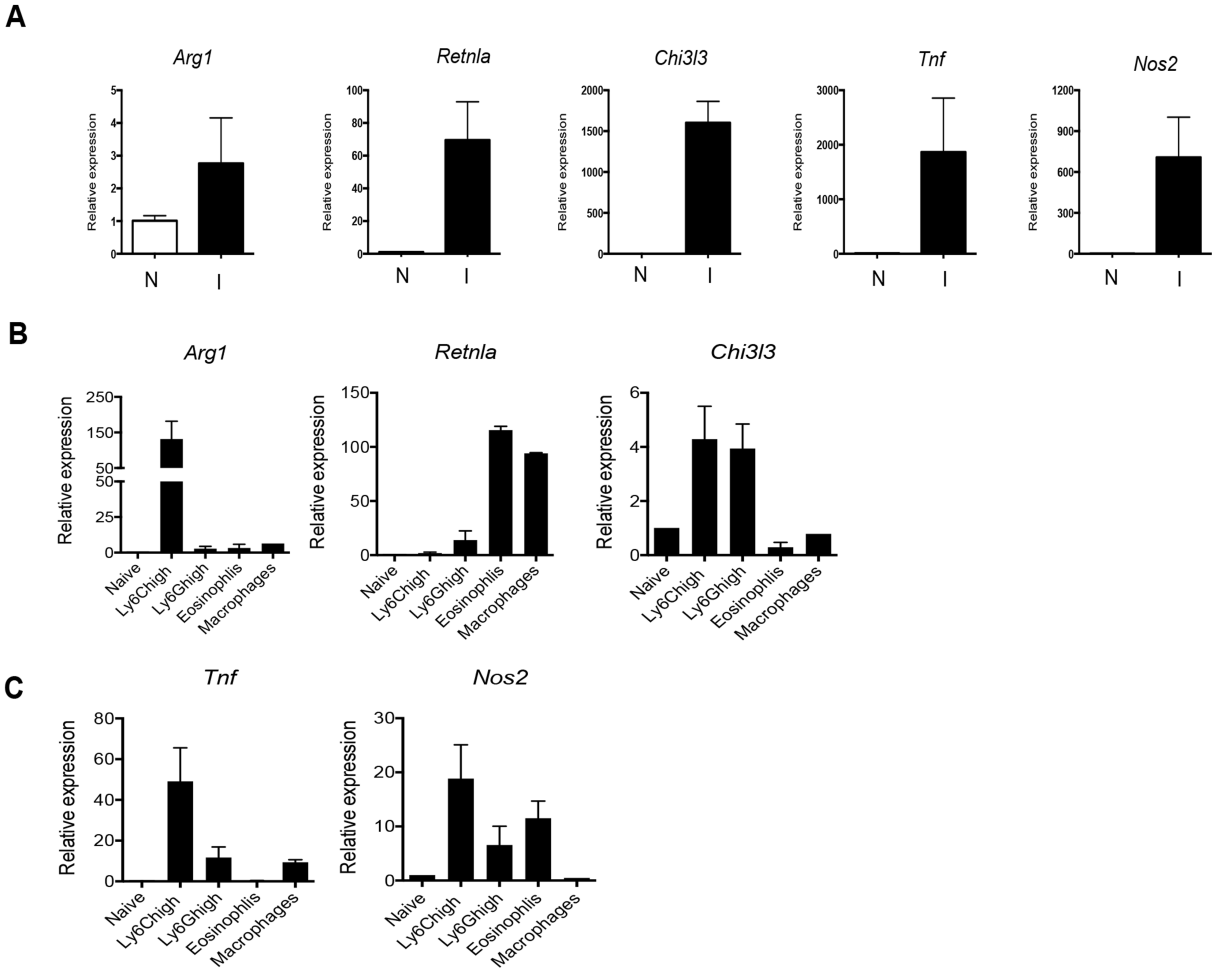
The M2 activation profile in the liver during infection results from differential expression of various M2 genes by distinct subpopulations of infiltrating leukocytes. Quantitative real-time PCR analysis of: **A**. *Arg1, Rtlna*, *Chi3l3*, *Tnf*, and *Nos2* mRNA in whole liver, comparing naïve (N) to infected (I) mice. **B**. *Arg1, Rtlna*, *Chi3l3* mRNA in the indicated cell populations, FACS-purified from livers of infected mice, presented as fold increase relative to expression in blood Ly6C^hi^ cells from naive mice. **C**. *Tnf*, and *Nos2* mRNA in cell populations as described in B. The data represent mean values ± SEM of data from 2 experiments, in each of which tissue or cells were pooled from 2 or more animals.

The fact that monocyte recruitment rather than in situ macrophage proliferation was primarily responsible for the increased hepatic macrophage population in infected mice led us to question the extent to which liver macrophages are M2 activated as a result of infection. We know that the M2-polarizing cytokine IL-4 is present within the hepatic milieu during infection [26], and previous reports indicate that hepatic macrophages are M2 activated in this setting [27], [28]. However, previous reports preceded the identification of stringent markers of macrophages [19]. Therefore, we sorted macrophages using MerTK and C64 expression and used real time RT-PCR to assess gene expression in these cells as well as in whole liver tissue. Using real time RT-PCR, we found that expression of the M2 markers Arginase-1 (*Arg1*), Relmα (*Retnla*), YM1 (*Chi3l3*), as well as the M1 markers TNFα (*Tnf*), and iNOS (*nos2*), was increased in liver from infected vs. naive mice (Fig. 8A), pointing to a complex array of signals that could favor monocyte recruitment to the liver even as IL-4 might also trigger macrophage proliferation. To determine which cell type(s) was responsible for expressing the genes under study, we sort-purified infiltrating cells based on the approach shown in Fig. 2A. We found that the overall M2 bias in the liver is accounted for by the expression of Relm-α in macrophages and eosinophils, Arginase-1 in monocytes, and YM-1 in monocytes and neutrophils (Fig. 8B). Our results for Relmα are consistent with previous reports, where macrophages and/or eosinophils have been shown to be major sources of this protein during schistosome egg associated inflammation [13], [15]. Thus, the overall M2 bias observed in the liver during acute infection is the result of a mosaic of gene expression in different cell types. Our gene expression analysis additionally revealed that Ly6C^hi^ monocytes in the liver of infected mice were expressing *nos2* and *tnf* (Fig. 8C).

## Discussion

Granulomatous inflammation rich in macrophages is common in helminth infections, when large parasite life stages become trapped in and or die within tissues during targeted or aberrant migration. While many features of helminth infection-associated granulomas are well documented, the origin of macrophages within them has remained relatively unclear. This question became particularly intriguing when it was reported that macrophages activated by IL-4, the quintessential product of Th2 cells which tend to dominate the immune response during helminth infections, undergo a proliferative response that can result in local population expansion. This process differs fundamentally from the recruitment of blood monocytes into tissues, and their subsequent differentiation into macrophages, which is considered to be the dominant source of macrophages associated with Th1 cell-associated inflammation in, for example, infections with bacteria or protists. During infection with *S. mansoni*, a strong Th2 response develops within the week following the initiation of egg production by mature female parasites and parasite eggs become trapped in the liver sinusoids and form focal points for intense CD4^+^ T cell dependent granulomatous inflammation. Because of the well-documented bias towards Th2 immunity in this system, and indeed the importance of IL-4 and of IL-4Rα expression by macrophages for the survival of mice infected with schistosomes, we hypothesized that macrophage activation within diseased liver tissue would be clearly M2 in nature (a notion supported by previous reports) and that local proliferation would therefore account for a significant component of the macrophage infiltrate. Our data reveal a different picture, in which the macrophage population exhibits signs of classical as well as alternative activation, and the recruitment of monocytes is the major process supporting the expansion of the macrophage population within the liver.

Our data clearly show a role for CCL2 in the recruitment of monocytes into liver tissue during infection with schistosomes. Levels of this chemokine were significantly elevated in the blood as a result of infection, and mice in which cells expressing the CCL2 receptor CCR2 were depleted had significantly diminished blood and liver monocyte numbers compared to infected WT mice. Our data are consistent with findings from previous reports that utilized a model system in which mice are injected intravenously with schistosome eggs, which embolize in the pulmonary vasculature and induce granulomatous inflammation. CCL2 is produced in the lungs in this model [29], and in mice that lack CCR2 the macrophage content of the lung granulomas that develop as a result of egg injection is initially reduced compared to that in egg injected WT mice [30]. Moreover, *ccl2-/-* mice were found to develop smaller granulomas in this model of egg induced granuloma formation [31]. Taking all of the findings together, it is clear that schistosome eggs induce CCL2 production and that CCR2/CCL2 play a role in monocyte recruitment during egg induced inflammation, and that monocytes recruited in this way are contributing to the expansion of the macrophage population at these sites. This is consistent with a role for CCL2 in increased macrophage numbers at sites of infections with other helminths [32], and with microbial pathogens [33], [34]. Monocytes can differentiate into macrophages in response to M-CSF [21] and into dendritic cells in response to GM-CSF [2], [35]. While we were able to detect M-CSF in the serum of infected mice, GM-CSF was undetectable (data not shown). However, it remains possible that GM-CSF is made within the hepatic environment of infected mice, and it will be interesting to examine dendritic cell differentiation from monocytes in this setting in the future.

Earlier work suggested that in addition to Ly6C^hi^ monocytes, Ly6C^lo^ monocytes are also recruited to the site of *L. monocytogenes* infection, via CX_3_CR1, and give rise to M2-like macrophages [36]. Our work argues that, in schistosomiasis, it is rather the Ly6C^hi^ monocytes that contribute to the pool of M2-like macrophages. In this regard, our findings are broadly consistent with recent findings that M2 macrophages in allergic skin are derived from inflammatory monocytes [37]. Our findings using CCR2-DTR mice revealed a critical role for monocytes for survival during acute schistosomiasis. While the system does not let us delineate between the importance of monocytes themselves and cells that differentiate from them, it was notable that in these animals, injection of DT resulted in loss of a subpopulation of CD11c^hi^ hepatic macrophages as well as of circulating and hepatic monocytes. Macrophages with this phenotype were present at significantly higher frequency following infection, and their loss in infected mice was evident histologically as significantly reduced hepatic granulomatous pathology, which nevertheless was associated with exacerbated disease. The latter is not altogether unexpected since granulomas are recognized to perform a host-protective function by sequestering egg hepatotoxins (discussed in [17]). Nevertheless, if this was the primary protective mechanisms disrupted by loss of CD11c^hi^ macrophages, we might have expected to see evidence of hepatocyte damage within the vicinity of parasite eggs, and this was not readily apparent. The lack of any difference in serum levels of the liver enzyme ALT between DT-treated WT and DT-treated CCD2-DTR mice supports the view that deletion of monocytes did not exacerbate liver damage in infected mice. It is possible that the major effect of monocyte-deletion during infection is in the intestine, although there was no gross evidence of severe intestinal damage or hemorrhage. Moreover, we failed to detect endotoxin in serum samples from DT treated infected WT or CCR2-DTR mice (<0.005 EU/mL, data not shown), which supports the view that intestinal integrity was maintained even when monocytes were acutely depleted (although a caveat of this conclusion is that we did not specifically test portal blood in this regard). We are also unclear why infected CCR2^−/−^ mice and DT-treated CCR2-DTR mice did not phenocopy each other. This lack of concordance is consistent with the development of a compensatory mechanism in the germline knockouts that provides the mice sufficient advantage to make it through acute infection. This could be related to the intense neutrophil infiltrate in the infected *Ccr2* ^−/−^ mice, which was not apparent in the DT-treated infected CCR2-DTR mice (data not shown). Understanding the difference in outcome of infection in these two strains of mice, along with the identification of the cause of death of infected mice acutely depleted of monocytes, awaits further investigation.

Recent work indicated that, in the steady state, liver macrophages are embryonically derived and genetically distinct from monocyte-derived cells and persist at the population level independently of replenishment from the bone marrow [38]. These macrophages are likely those that are CD11c^lo^ and which resist deletion by DT treatment in CCR2-DTR mice (although an alternative possibility is that these cells are derived from monocytes prior to the initiation of DT treatment). This population responds to infection by increased expression of MHCII and proliferation, but its exact function in the context of schistosomiasis remains unknown at this time. We suspect that it may play an important role, because broad depletion of macrophages using clodronate liposome injection at 7 weeks of infection caused a significantly more rapid onset of lethal disease than did DT administration to CCR2-DTR mice, such that all treated animals (n = 15) died within 24 h (data not shown; clodronate treatment of naïve mice caused no noticeable ill effects). A tentative conclusion from these results is that the CD11c^lo^ macrophages cooperate with the CD11c^hi^ macrophages in a host-protective role during schistosome infection.

One protective effect of M2 macrophages in schistosomiasis is to regulate the intensity of Th2 immune responses. This is critical in schistosomiasis, where unregulated Th2 responses can lead to excessive and sometimes lethal immunopathology. Arginase 1 and Relmα, both of which are considered to be canonical markers of M2 activation, have been shown to play roles in these processes, although their importance appears to be greater at times later in infection than those tested here [14], [15]. Nevertheless, it is possible that the marked effects of deletion of CCR2 cells that we observed here reflect the loss of monocytes or macrophages capable of making Arginase1 and Relmα. Of interest in this regard, we observed a trend towards increased production of IL-4, IL-5 and IL-13, and decreased production of IL-10 by splenocytes from infected DT treated CCR2-DTR mice, which is consistent with the loss of mechanisms that regulate Th2 cells. Our failure to detect IL-4 in the supernatants of SEA-stimulated splenocytes from infected WT mice is likely due to the consumption within the cultures of this cytokine, since in previous reports we have shown that the addition of blocking anti-IL-4Rα antibodies allows subsequent accumulation of IL-4 to measurable levels. A corollary of this is that CCR2^+^ cells are precisely those responsible for consuming IL-4. This possibility will be addressed in future studies.

Our data support the view that there is a conflict of pro- and anti-inflammatory events occurring within the liver during infection, and suggest that the overall pattern of immune gene expression in this organ reflects the involvement of different cellular populations within the infiltrate. Interestingly, both NO and TNFα, which we found to be made by infiltrating monocytes during infection, are implicated in severe morbidity and increased mortality due to schistosomiasis in the absence of IL-4 [17], raising the possibility that it is unregulated inflammatory monocyte invasion and activation that underpins this condition. Whether individual monocytes express Arginase1, iNOS and TNFα, or whether the RT-PCR data reflect the existence within the monocyte gate of cells committed to the production of one, or more than one of these mediators, is unknown at present. These questions are being addressed in ongoing research.

The difficulty of correlating findings from *in vitro* macrophage polarization experiments and *in vivo* infections has been addressed previously [39], and our findings support this view. Despite the well-documented dominance of the Th2 response during schistosomiasis [17], it is clear that in this infection macrophages do not conform fully to the M2 phenotype observed in IL-4 stimulated bone marrow derived macrophages [7], [40]. This likely reflects the impact of other cytokines, such as IFNγ, which may modulate the M2 phenotype during infection [15]. Indeed, the presence of iNOS and TNFα expressing cells in the liver may reflect the superimposition of an inflammatory signal arising from the leakage of TLR agonists from the intestinal flora into the portal bloodstream as schistosome eggs follow their natural route of egress from the vasculature into the gut lumen, perforating the epithelium *en route* [17]. In this way, schistosomiasis differs from the “sterile” nematode infection or implantation models in which resident macrophage proliferation in the absence of monocyte recruitment has been documented [5], [41].

## Materials and Methods

### Ethics statement

All work was conducted in strict accordance with the recommendations in the Guide for the Care and Use of Laboratory Animals of the National Institutes of Health. The protocol was approved by the Institutional Animal Care and Use Committee of Washington University in St. Louis School of Medicine (Animal Welfare Assurance Number: A-3381-01).

### Animals and parasites

C57BL/6J, B6.129P-*Cx_3_cr1^tm1 Litt^*/J (*Cx_3_cr1^gfp/gfp^*), B6.129S4- *Ccr2^tm1Ifc^/J* (*Ccr2^−/−^*), male mice (all from Jackson Laboratories) were used in this study. B6 CCR2-DTR mice were a gift from Dr. Eric Pamer, and were bred at Washington University. For experiments using these mice, CCR2-DTR heterozygotes were compared with WT littermates. *Cx_3_cr1^gfp/+^* mice were obtained by breeding *Cx_3_cr ^gfp/gfp^* mice with C57BL/6J mice. *Cx_3_cr1 ^gfp/+^* mice have one *Cx_3_cr1* allele replaced with cDNA encoding *Egfp*, and can be used to track monocytes [22]. Mice were kept under specific pathogen–free conditions and were infected at 8–12 weeks of age. Mice were each percutaneously exposed to 150 *Schistosoma mansoni* cercariae. Snails were provided by the Schistosome Research Reagent Resource Center for distribution by BEI Resources, NIAID, NIH: *Schistosoma mansoni*, Strain NMRI Exposed *Biomphalaria glabrata*, Strain NMRI, NR-21962. Uninfected mice were used as controls. Mice were euthanized at 7–9 weeks post infection.

### Tissue histology

Tissues were fixed in neutral buffered formalin, embedded in wax, sectioned and stained with hematoxylin and eosin. Slides were initially read and interpreted in a blinded fashion. The diameters of granulomas (around single eggs) were measured in a horizontal plane bisecting central eggs using the LAS imaging program (Nikon).

### Cytokine, chemokine, endotoxin and ALT measurements

CCL2, CCL7, M-CSF and GM-CSF levels in plasma pools from 5 uninfected or 5 infected mice were measured using the Rodent MAP v2.0 by RBM-Myriad. Cytokines in splenocyte supernatants were measured by cytometric bead array (CBA, BD). Bacterial Gram negative endotoxin in serum samples was measured using the Chromo Limulus Amebocyte Lysate assay (Associates of Cape Cod Inc.). ALT in serum samples was detected using a coupled enzyme assay to colorimetrically measure enzyme activity (Sigma-Aldrich).

### Cells

Peripheral blood was drawn via cardiac puncture with heparin. Erythrocytes were removed by incubating with red blood cell lysis buffer. Bone marrow was flushed out of femurs using RPMI. To analyze hepatic cell populations livers were removed from PBS-perfused animals, crushed with a syringe plunger, and incubated in RPMI (Hyclone) containing 250 µg/ml of Collagenase D (Roche) at 37°C for 60 min. The resulting suspension was disrupted through a 100 µm metal cell strainer and centrifuged at room temperature for 20 min at 2500 RPM through 40% isotonic Percoll/RPMI. The resulting pellet was washed, and used for analysis. Splenocytes were prepared and cultured alone or with soluble egg antigen (SEA, 50 µg/ml), as previously described [42].

### Diphtheria toxin treatment

Diphtheria toxin (DT) was from List Biological Laboratories (Cat. No. 150), reconstituted at 1 mg/ml in PBS, and frozen at −80°C. Mice received 10 ng/g DT via the i.p. route in 0.2–0.3 ml PBS [25].

### Clodronate treatment

Mice were each treated by i.v. injection of 250 µl of liposomes containing clodronate (clodronateliposomes.com).

### In vivo BrdU pulse-chase

Mice were injected (i.p.) with 1.0 mg BrdU (BD Biosciences) dissolved in PBS at 4 hours, 24 hours, 4 days, and 7 days before experimental end-points. Cells were prepared as described above and the BrdU in situ detection Kit (BD Bioscience) was used according to the manufacturer's instructions to stain for BrdU incorporation.

### Flow cytometry

Samples were blocked with 5 µg/mL of anti-CD16/CD32 (eBioscience) and incubated with combinations of the following antibodies, which were either directly conjugated to fluors, or biotinylated: anti-Ly6C (HK 1.4, BD Biosciences); anti-Ly-6G (1A8, BD Biosciences); anti-CD11b (M1/70, BD Biosciences); anti-F4/80 (BM8; eBioscience); anti-CD11c (HL3, BD Biosciences); anti-I-A/I-E (M5/114.15.2, Biolegend); anti-CD115 (AFS98, Biolegend); anti- CD45 (30F11, Biolegend); anti-CD64 (X54-5/7.1,BD Bioscience); anti-mMer (MerTK, R&D Systems); anti-Siglec F (E50-2440, eBioscience); Ki67 (B56, BD Biosciences, used in conjunction with elements of the BrdU detection kit for cell permeabilization). Strep-PECy7 (BD Biosciences) was used to detect biotinylated antibodies. Cells were stained with LIVE/DEAD (Invitrogen) before analysis. Data were acquired on a Canto II (BD Biosciences) and analyzed with FlowJo v.8.8.6 (Tree Star, Inc.). Cells were sorted on a BD FACSAria (BD Biosciences). For morphologic characterizations, sorted cells were prepared on slides by cytocentrifugation at 1000 RPM for 5 min, and stained with HEMA-3 (Fischer Scientific).

### In vitro macrophage differentiation

FACS-sorted Ly6C^hi^ monocytes from blood and livers were treated with M-CSF (0.02 µg/mL) in RPMI (Hyclone) supplemented with 10% FCS (Hyclone), 50 µM 2-Mercaptoethanol (Cellgro, Mediatech, Inc., Va) and 100 U/mL Penicilin-Streptomycin (Cellgro, Mediatech, Inc., Va). Cells (10^5^) were plated in triplicate in 96-well round bottom plate (Costar, Corning Inc., NY) and cultured in a humidified incubator at 37%, 5% CO2 [21]. Cells were harvested at day 7, and the expression of the surface marker F4/80, CD64 and MertK were determined.

### RNA isolation and quantitative RT PCR

Classical monocytes, neutrophils, eosinophils and macrophages were sorted from the leukocyte populations isolated from livers pooled from 5–10 naïve or infected mice. Pieces of liver were also used for RNA isolation. Cells or liver samples were resuspended in 1.0 mL Trizol (Invitrogen) and RNA was extracted using the manufacturer's instructions. Qiagen's RNeasy Micro “RNA clean-up” protocol with an on-column DNase treatment was used to purify the RNA further. RNA yield and integrity were assessed with a NanoDrop spectrophotometer (Thermo-Scientific). PCR cDNA was generated from 500 ng of RNA per sample by reverse transcription (RT). First strand cDNA was synthesized using isolated RNA, Superscript II reverse transcriptase (Invitrogen), and oligo dT as a primer. Relative quantification of the genes was performed by Applied Biosystems 7500 real-time PCR system with Taqman Gene Expression Master mix (Invitrogen) and the 2^-ΔΔCt^ analysis. Total reaction volume was 20 µL with 300 nM of each primer/probe, 10 µl of master mix, and 1 µl of cDNA as template (or water as a negative control). Expression of *Arg1* (Mm00475988_m1), *Retnla* (Relmα; Mm00445109_m1), *Chi3l3* (YM1; Mm00657889_s1), *tnf* (Mm00443260_g1) and *Nos2* (Mm00440502_m1), were normalized to β-actin, *Actb* (Mm00607939_m1).

### Statistics

Results are expressed as mean ± SEM, or ± SD. Statistical tests included unpaired, 2-tailed Student's *t* test. *P* values of 0.05 or less were considered to denote significance.
